# Indents

**Published:** 1909-01-15

**Authors:** 


### INDENTS.

☞ **Brief Articles of Technical or Scientific Value will receive notice and comment. Contributions invited.**

*Aids in Taking Impressions.* This clinic consisted in producing an easy and definite line of fracture in all plaster impressions, either in orthodontia or prosthetic work. The clinician uses the Angle pattern tray for all work. For taking a full upper or lower impression for orthodontia work, a tray of sufficient size is selected.

Trays for plaster impressions should always be kept bright and highly polished. This is not a difficult task if, upon removal from the mouth, the tray is at once immersed in boiling water and all plaster wiped off and the tray polished with a soft cloth and placed in a flannel pocket. This can be done before the plaster is removed from the mouth. The tray if bright readily separates from the plaster, and this gives the plaster time to harden thoroughly. To produce this definite line of fracture in the plaster, cut a piece of wax about three sixteenths of an inch wide from the long side of a sheet of wax, stand it on edge in the trough of the tray, conforming in shape to the outline of the arch. This is secured in position by a hot spatula or by warming the tray in a dry flame. Another piece about the same width is placed in the median line and attached to the labial rim in the same manner. With a warm spatula bevel the upper edge of the wax to a knife edge-to prevent a broad line of fracture should it come in contact with the teeth. The plaster is now placed in the trough of the tray, leaving the palatal portion free, in case of uppers, and with a spatula the vault is filled to insure the exclusion of all air and to prevent bubbles. The cup is now placed in position, whereby care is taken not to press it up too far. It is better not to have the wax come quite in contact with the teeth. The surplus plaster will fill the space below the plaster in the vault and if reasonable care has been taken, no plaster will run back into the throat. The cup is held in position until the patient feels the warmth from the setting of the plaster. The cup may now be removed and this is done by firmly pressing downward on the handle, when it will readily separate, leaving the plaster in the mouth. while the plaster is further hardening, clean the cup and put it away as described. The object of the wax is now plainly seen. With a wax spatula or some other flat instrument insert it in the wax in the median line, and by using the instrument as a lever, first in one direction and then in the other, each lateral half or side will fracture away, and the palatal portion can readily be removed by inserting the instrument in the plaster and using the edge of the teeth as a fulcrum. The lower impression is taken in the same manner, except that a piece of wax is placed on both sides of the arch in the median line. The parts are now washed, dried, and stuck together with wax, after which they are given one coat of sandarac varnish, which, if placed at the right time, is sufficient to give a good gloss. Ten or fifteen minutes is about the right time, for if an impression is allowed to stand longer it becomes dry and the varnish is absorbed and leaves a dull surface which will not separate. Never use soap or oil under any circumstances if you wish hard white models free from fine bubbles. If the labial and buccal rims of the impression are not uniform, it is best to make them so by building them out with wax. A uniform rim is necessary in order to have a uniform roll on the model. and it also acts as a guide in shaping up the plaster while it is hardening. Remember always that it is easier to cut soft than hard plaster.—Dr. M. L. Fay, in *Cosmos*.

*Swaging Rubber Plates.* The method of swaging rubber plates as advocated by Dr. Linet is not only very practical, but affords the construction of a well-fitting plate of uniform thickness, whose palatal side faithfully reproduces the rugae. The generally adopted use of rubber offers two great disadvantages:

(1) It irritates the mucous membrane and sometimes even causes abscess. This is due to its poor conductivity of heat and to the usually excessive thickness of the base-plate.

(2) The massive construction and the difficulty in obtaining a uniform thickness often involves loss of taste and impediment in speech.

To do away with the excessive thickness and the want of natural forms, the author does not model and smooth his plate on the lingual surface, but stamps it like a metal plate. After securing a model as for a gold plate, the following procedure is adopted:

(1) From the plaster model a zinc die and counter-die in lead are made.

(2) With tin foil of the thickness used in making the patterns from which metal plates are cut, a base-plate is made on the zinc model which takes the place of the wax in an ordinary appliance. This base-plate is obtained by burnishing several tin-foil sheets over the zinc.

(3) After a layer of five or six sheets has been obtained, the plate is lightly swaged with the counter-die, after placing a piece of fine linen between in order to avoid sticking.

(4) After the plate thus obtained has been trimmed, it is again swaged as before, only more strongly. It is then adjusted to the plaster model, and the rest of the appliance is constructed like an ordinary rubber plate. The packing of the plaster model requires great care, and the counter-die must be flowed perfectly in order to reproduce on the rubber all the details of the plate. After the plaster has hardened, the wax is melted and the tin foil carefully removed. Then the rubber is packed as usual, and the die is tightly closed in order to avoid any uneven thickness.

The rubber plate is vulcanized by gradually raising the heat and keeping it at an even temperature of 320 for one hour and a half. If, in polishing, the exact form of the tin foil is preserved, the plate will surpass a metal plate as well as an ordinary rubber plate. The disagreeable sensation of heat is done away with by this rubber plate with a metallic base, the food particles do not stick as firmly as they would to a rubber plate, and the elasticity of the latter, which is one of its advantages over a metal plate, is preserved.—Dr. E. Linet in Dec. 1908 *Cosmos.*

